# Nrf2-Inducing Anti-Oxidation Stress Response in the Rat Liver - New Beneficial Effect of Lansoprazole

**DOI:** 10.1371/journal.pone.0097419

**Published:** 2014-05-20

**Authors:** Yasunobu Yamashita, Takashi Ueyama, Toshio Nishi, Yuta Yamamoto, Akatsuki Kawakoshi, Shogo Sunami, Mikitaka Iguchi, Hideyuki Tamai, Kazuki Ueda, Takao Ito, Yoshihiro Tsuruo, Masao Ichinose

**Affiliations:** 1 2nd Department of Internal Medicine, Wakayama Medical University Graduate School of Medicine, Wakayama, Japan; 2 Department of Anatomy and Cell Biology, Wakayama Medical University Graduate School of Medicine, Wakayama, Japan; Nihon University School of Medicine, Japan

## Abstract

Lansoprazole is a potent anti-gastric ulcer drug that inhibits gastric proton pump activity. We identified a novel function for lansoprazole, as an inducer of anti-oxidative stress responses in the liver. Gastric administration of lansoprazole (10–100 mg/kg) to male Wistar rats produced a dose-dependent increase in hepatic mRNA levels of nuclear factor, erythroid-derived 2, -like 2 (Nrf2), a redox-sensitive transcription factor, at 3 h and Nrf2 immunoreactivity (IR) in whole hepatic lysates at 6 h. Conversely, the levels of Kelch-like ECH-associated protein (Keap1), which sequesters Nrf2 in the cytoplasm under un-stimulated conditions, were unchanged. Translocation of Nrf2 into the nuclei of hepatocytes was observed using western blotting and immunohistochemistry. Expression of mRNAs for Nrf2-dependent antioxidant and phase II enzymes, such as heme oxygenase 1 (HO-1), NAD (P) H dehydrogenase, quinone 1 (Nqo1), glutathione S-transferase A2 (Gsta2), UDP glucuronosyltransferase 1 family polypeptide A6 (Ugt1a6), were dose-dependently up-regulated at 3 h. Furthermore, the levels of HO-1 IR were dose-dependently increased in hepatocytes at 6 h. Subcutaneous administration of lansoprazole (30 mg/kg/day) for 7 successive days resulted in up-regulation and nuclear translocation of Nrf2 IR in hepatocytes and up-regulation of HO-1 IR in the liver. Pretreatment with lansoprazole attenuated thioacetamide (500 mg/kg)-induced acute hepatic damage via both HO-1-dependent and -independent pathways. Up-stream networks related to Nrf2 expression were investigated using microarray analysis, followed by data mining with Ingenuity Pathway Analysis. Up-regulation of the aryl hydrocarbon receptor (AhR)-cytochrome P450, family 1, subfamily a, polypeptide 1 (Cyp1a1) pathway was associated with up-regulation of Nrf2 mRNA. In conclusion, lansoprazole might have an alternative indication in the prevention and treatment of oxidative hepatic damage through the induction of both phase I and phase II drug-metabolizing systems, i.e. the AhR/Cyp1a1/Nrf2 pathway in hepatocytes.

## Introduction

Lansoprazole is a potent proton pump inhibitor that reduces the secretion of gastric acid from gastric parietal cells by inhibition of H^+^/K^+^-adenosine triphosphatase. It has been shown that lansoprazole is effective for the treatment and prevention of a broad range of acid-related diseases such as gastro-esophageal reflux disease (GERD), duodenal and gastric ulcers and non-ulcer dyspepsia [1], [2]. Recent studies have shown that lansoprazole has acid-independent protective effects in the gastrointestinal mucosa, such as anti-inflammatory effects and anti-bacterial effects on *Helicobactor pylori* [3]. Both capsaicin-sensitive sensory neurons and nitric oxide are involved in the acid-independent gastrointestinal protective effects of lansoprazole [4]. Induction of the antioxidant defense enzyme, heme oxygenase-1 (HO-1) in human endothelial and gastric cancer cells, rat gastric epithelial cells (RGM-1), or the epithelium of small intestine is also involved in acid-independent gastrointestinal mucosal protection [5]–[7]. These reports demonstrated that lansoprazole, but not omeprazole, induced HO-1 in gastric mucosal cells [6] and the small intestine [7]. Therefore, we used lansoprazole to investigate anti-oxidative stress responses in the liver.

HO-1 is a highly inducible, stress-responsive protein (also called heat shock protein 32), which catalyzes the first and rate-limiting step in heme degradation to produce equimolar quantities of biliverdin, carbon monoxide (CO) and free iron [8]. Heme is a potent oxidant, while bilirubin converted from biliverdin and CO exhibits antioxidant activity, vasodilation and inhibition of platelet aggregation, respectively. Therefore, induction of HO-1 can provide cytoprotection against oxidative stress.

Induction of HO-1 is controlled by a redox-sensitive transcription factor, nuclear factor, erythroid-derived 2, -like 2 (Nrf2). Nrf2 is a master transcription factor that regulates antioxidant response element (ARE)-mediated transcription of genes involved in the regulation of the synthesis and conjugation of glutathione (glutamate-cysteine ligase catalytic subunit), antioxidant proteins specializing in the detoxification of certain reactive species (HO-1), drug-metabolizing enzymes (UDP-glucuronosyl-transferase 1A1), xenobiotic transporters (multidrug resistance protein 1), and molecular chaperones [9]–[11]. Nrf2 is sequestered in the cytoplasm by Kelch-like ECH-associated protein (Keap1) under un-stimulated conditions, while Nrf2 is translocated into the nucleus and activates the electrophilic response element/antioxidant response element (EpRE/ARE) upon exposure to oxidative insults [9]–[11]. It was shown that significant amounts of the Nrf2-Keap1 complex remained in the bound form after exposure to electrophiles [12], [13]. The two mechanisms that have been proposed for Keap1-Nrf2 dissociation are phosphorylation of Nrf2 [14] and modification of Keap1 [12]. Sulfhydryl groups in Keap1 cysteine residues are the main targets of oxidation and electrophilic modification [12]. Modulation of the Keap1/Nrf2/ARE system is a potential pharmacological target for ameliorating oxidative stress. Activators and inhibitors of the Keap1/Nrf2/ARE system include endogenous substances formed in cells/tissues, such as reactive oxygen species (ROS), hydrogen sulfide, lipid peroxidation products, hormones and neurotransmitters (15-deoxy-Δ^12, 14^ -prostaglandin J_2_, catechol estrogens and dopamine), as well as exogenous substances derived from food, air, or other sources (medical procedures, radiation, UV irradiation) [15]. Within exogenous inducers, sulfur-containing glucosinolates derived from cruciferous vegetables (broccoli, Brussels sprouts, horseradish, etc.) are well known [16]. Sulforaphane derived from cruciferous vegetables induces ROS formation through auto-oxidation or disruption of the mitochondrial respiration chain [13]. Most of the chemicals reported to activate this system depend on the dissociation of Nrf2 from Keap1. Few chemicals induce and increase the levels of free Nrf2. It was proposed that increased synthesis of Nrf2 could be a mechanism underlying the Nrf2-activating effects of α-lipoic acid [17]. Mixtures of plant extracts, such as “Protandim” (LifeVantage, USA) and “Nrf2 activator” (XYMOGEN, USA) are available as inducers of the Keap1/Nrf2/ARE system. However, these plant extracts have a very wide range of biological effects, making it difficult to distinguish the true contribution of Nrf2 induction. Currently, more specific and effective synthetic chemicals are being developed [11]. In this study we report that lansoprazole is a strong and effective inducer of Nrf2 transcription in hepatocytes, in addition to acting as a proton pump inhibitor. Lansoprazole up-regulated the levels of Nrf2 mRNA and IR without affecting the levels of Keap1 mRNA and IR, thereby promoting the translocation of unbound Nrf2 into the hepatic nuclei.

The metabolism of chemicals and drugs involves a series of successive enzymatic reactions [18]. First, in phase I reactions, reactive or polar groups are introduced to the chemicals by a superfamily of cytochrome P450 oxidases (CYPs) such as cytochrome P450, family 1, subfamily a, polypeptide 1 (Cyp1a1), and family 1, subfamily b, polypeptide 1 (Cyp1b1), followed by phase II reactions mediated by detoxifying and antioxidant enzymes. Phase II drug-metabolizing enzymes, such as glutathione S-transferase and UDP glucuronosyltransferase, transfer and conjugate hydrophilic side chains to polar groups. Nrf2 mediates the transcription of mRNAs for phase II enzymes, while the aryl hydrocarbon receptor (AhR) mediates the transcription of phase I enzymes [18]. Lansoprazole also up-regulates the mRNA levels of AhR and Cyp1a1 in the liver. We report for the first time that lansoprazole up-regulates the AhR/Cyp1a1/Nrf2 pathway in hepatocytes and has a potential application in the prevention and treatment of oxidative hepatic damage.

## Materials and Methods

### Ethics Statement

The Wakayama Medical College Animal Care and Use Committee approved all animal manipulations (No 543, No 566 and No 572).

### Tissue preparation

Male Wistar rats, 6 weeks old, were purchased from Kiwa Laboratory Animals Co., Ltd. (Wakayama, Japan). The rats were housed in a temperature-controlled environment. Experiments were performed after providing the rats with free access to food and water for 1 week. The rats were fasted overnight prior to gastric administration of drugs in individual wire-bottom cages. Lansoprazole (supplied by Takeda Pharmaceutical Co., Ltd., Osaka, Japan) was suspended in 0.5% methylcellulose. The rats were intra-gastrically administered (by gastric intubation) lansoprazole 10 mg/kg, 30 mg/kg, 100 mg/kg or vehicle (n = 10). The rats were decapitated at 3 h and 6 h after administration of lansoprazole or vehicle. The liver was rapidly removed, and several pieces were immediately frozen (within 1 min after decapitation) using powdered dry ice. The rest of the liver was fixed in 4% paraformaldehyde in 0.1 M phosphate buffer (pH 7.4) overnight at 4°C, then cryo-protected in phosphate-buffered saline (PBS) containing 30% sucrose for 3 days at 4°C. The tissue samples were mounted in O.C.T. compound (Tissue-Tek, Sakura Finetek Japan Co., Ltd., Tokyo, Japan) and frozen using powdered dry ice. The frozen samples were stored at −80°C until sectioned and assayed.

In the second experiment, the rats were subcutaneously (s.c.) administered lansoprazole 30 mg/kg/day for 7 successive days. The rats were decapitated 1 day after the final administration of lansoprazole (n = 5) and the livers were sampled as described above. Other groups of rats (n = 5) were administrated vehicle (s.c., 0.5% methylcellulose) for 7 successive days.

### Acute hepatic injury model and pharmacological treatment with stannous mesoporphyrin

Other groups of rats were administered lansoprazole (s.c., 30 mg/kg/day) or vehicle (s.c., 0.5% methylcellulose) for 7 successive days. The rats were intra-peritoneally (i.p.) administrated thioacetamide (TAA; 500 mg/kg) [19] and 48 h after TAA treatment, an intracardiac blood collection was performed under anesthesia with medetomidine hydrochloride (0.15 mg/kg), midazolam (4 mg/kg) and butorphanol tartrate (5 mg/kg), and the serum was obtained. The liver was also rapidly removed, and fixed as described above.

Hepatic damage was assessed by measuring serum aspartate aminotransferase (AST) and alanine transaminase (ALT) activities according to the standard methods (SRL, Inc., Tokyo, Japan). Hepatic damage was also assessed by histological examination with hematoxylin-eosin staining. The area of hepatocelluar degeneration/necrosis in the section was assessed with the aid of Image J (http://imagej.nih.gov/ij/). In short, the digitized images were transferred to a personal computer, and the border of the lesion and the total area of the liver in the section were traced on a computer by a single observer who was blind to the treatments. The injured area per total liver area was calculated as lesion index (%). Stannous mesoporphyrin (SnMP, BIOMOL Research Labs. Inc., Plymouth Meeting, PA, USA), an HO-1 inhibitor, was dissolved in 100% ethanol and diluted 10-fold in 7% NaHCO3. The rats received vehicle or SnMP (20 µmol/kg) intra-peritoneally 60 min before administration of TAA [20].

The rats were divided into six groups. Rats in Group A (n = 5) (control) received daily (8AM) s.c. administration of vehicle for 5 successive days. At noon on the 4^th^ day, the rats received i.p. administration of vehicle, followed by a second i.p. injection of vehicle. Rats in Group B (n = 5) (lansoprazole) were administered lansoprazole (s.c., 30 mg/kg/day) daily (8 AM) for 5 successive days. At noon on the 4^th^ day, the rats received i.p. administration of vehicle, followed by a second i.p. injection of vehicle. Rats in Group C (n = 4) (acute hepatic damage) were administered vehicle (s.c.) daily (8 AM) for 5 successive days. At noon on the 4^th^ day, the rats received i.p. administration of vehicle, followed by i.p. injection of TAA. Rats in Group D (n = 4) (lansoprazole and acute hepatic damage) were administered lansoprazole (s.c., 30 mg/kg/day) daily (8 AM) for 5 successive days. At noon on the 4^th^ day, the rats received i.p. administration of vehicle, followed by i.p. injection of TAA. Rats in Group E (n = 4) (HO-1 inhibitor and acute hepatic damage) were administered vehicle (s.c.) daily (8 AM) for 5 successive days. At noon on the 4^th^ day, the rats received i.p. administration of SnMP (20 µmol/kg), followed by i.p. injection of TAA. Rats in Group F (n = 4) (lansoprazole, HO-1 inhibitor and acute hepatic damage) were administered lansoprazole (s.c., 30 mg/kg/day) daily (8 AM) for 5 successive days. At noon on the 4^th^ day, the rats received i.p. administration of SnMP (20 µmol/kg), followed by i.p. injection of TAA. Forty-eight hours after TAA treatment, intracardiac blood collection was performed under anesthesia with medetomidine hydrochloride (0.15 mg/kg), midazolam (4 mg/kg) and butorphanol tartrate (5 mg/kg), and both serum and liver tissue samples were obtained.

### Extraction of total RNA

Total RNA from livers was extracted using the RNeasy Mini Kit (QIAGEN, Tokyo, Japan) and digested with RNase free-DNase (QIAGEN). Using a NanoDrop 1000 (Thermo Fisher Scientific Inc., Shanghai, China), the 260∶280 nm absorbance ratio (A260/280) and the 260∶230 nm absorbance ratio (A260/230) of the RNA samples were measured. In this experiment, we used RNA samples with A260/280 ratios greater than 1.8 and A260/230 ratios greater than 1.5. The quality of purified RNAs was assessed using an Agilent 2100 Bioanalyzer with an RNA 6000 Nano Kit (Agilent Technologies, Palo Alto, CA, USA).

### Real-time RT-PCR

Expression of mRNA was determined using real-time reverse transcription (RT)-polymerase chain reaction (PCR). Primer sets for each gene are listed in Table 1. As an internal control, we also estimated the expression of rat glyceraldehyde-3 phosphate dehydrogenase (GAPDH) mRNA. Total RNA (0.1 µg) was converted into cDNA by reverse transcription using random primers (p (dN)_ 6_ primers) and AMV reverse transcriptase (Roche Diagnostics Corp., Indianapolis, IN, USA) in a total reaction volume of 20 µl. PCR amplification using a LightCycler instrument was carried out in 20 µl of reaction mixture consisting of LightCycler FastStart DNA Master SYBR Green I (Roche Diagnostics GmbH, Penzberg, Germany), 4.0 mM of MgCl_2_, 0.5 µM of each probe, and 2 µl of template cDNA in a LightCycler capillary. Relative mRNA levels in each sample were quantified automatically according to the standard curves constructed according to the LightCycler software. The levels of mRNA were calculated with reference to external standard curves constructed by plotting the log number of 10-fold serially diluted cDNA samples against the respective threshold cycle using the second derivative maximum method. Expression of mRNA levels in each sample was normalized to GAPDH mRNA levels.

**Table 1:** List of oligonucleotide primers used for RT-PCR.

Gene	Accession number	Forward primer	Reverse primer
Glyceraldehydes-3 phosphate dehydrogense	NM_017008	AGGTTGTCTCCTGTGACTTC	CTGTTGCTGTAGCCATATTC
Nuclear factor, erythroid derived 2, like 2 (Nfe2l2)(Nrf2)	NM_031789	CACATCCAGACAGACACCAGT	CTACAAATGGGAATGTCTCTGC
Kelch-like ECH-associated protein 1 (Keap1)	NM_057152	GGACGGCAACACTGATTC	TCGTCTCGATCTGGCTCATA
Heme oxygenase 1 (HO-1)	NM_012580	ACAGGGTGACAGAAGAGGCTAA	CTGTGAGGGACTCTGGTCTTTG
Glutathione S-transferase A2 (Gsta2)	NM_017013	CTTCTCCTCTATGTTGAAGAGTTTG	TTTTGCATCCACGGGAA
NAD(P)H dehydrogenase, quinone 1 (Nqo1)	NM_017000	CAGCGGCTCCATGTACT	GACCTGGAAGCCACAGAAG
UDP glucuronosyltransferase 1 family, polypeptide A6 (Ugt1a6)	NM_001039691	ACTCAAAGTATGAGATCCTTGC	TCAAATTCCTGAGACAGGTTC
aryl hydrocarbon receptor (AhR)	NM_013149.2	CAG GCG TTC CTA AGC AAG TTT C	GGA GGT GAG CAG CAG TCT GA
cytochrome P450, family 1, subfamily a, polypeptide 1 (Cyp1a1)	NM_012540.2	GTG GCC TGT ATT TTG CTT ATG	AGC TCA GGT ACG TTT TTC CTA
peroxisome proliferator activated receptor alpha (Pparα)	NM_017232.3	CAG CCA CCA TCA ACG CAA GT	TTA CAG CTC AGT TGA ACG CCT TTT G

### Western blotting

Frozen liver tissues were minced, homogenized in a buffer containing 0.01 M Tris-HCl, pH 7.6, 0.15 M NaCl, 1% TritonX-100, and protease inhibitor cocktail (0.2 mM phenylmethanesulfonyl fluoride, 20 µM leupeptin and 1.5 µM pepstatin A). The homogenates were centrifuged at 10,000 x g for 15 min at 4°C. Protein concentrations were determined using a Bio-Rad Protein Assay kit (Bio-Rad Laboratories Inc., Hercules, CA, USA).

The samples were subjected to SDS-polyacrylamide gel (12.5%) electrophoresis and subsequently immunoblotted. After blocking with skim milk (5%), the blots were incubated overnight at 4°C with primary antibodies diluted in TBST (0.01 M Tris-HCl, pH 7.6, 0.15 M NaCl, 0.05% Tween 20) containing 1% BSA, as follows: anti-Nrf2 (1.0 µg/ml; rabbit polyclonal, ab137550, Abcam, Tokyo, Japan), anti-Keap1 (1.0 µg/ml; goat polyclonal, AF3024, R&D Systems Inc., Minneapolis, MN, USA), anti-HO-1 (1∶20,000; rabbit polyclonal, SPA-895, Stressgen Biotechnologies Corp., Victoria, Canada), anti-β-actin (1∶200; rabbit polyclonal, A5060, Sigma, St. Louis, MO, USA), anti-Calpain (1∶200; rabbit polyclonal, H-240, Santa Cruz Biotechnology Inc., Santa Cruz, CA, USA), anti-Histone H1 (1∶200; rabbit polyclonal, FL-219, Santa Cruz), respectively. After washing in TBST, the blots were incubated with peroxidase-conjugated goat anti-rabbit antibody (1∶5000; NA934, GE Healthcare UK Ltd, Amersham Place, Little Chalfont, Buckinghamshire, HP7 9NA, England) or with peroxidase-conjugated donkey anti-goat antibody (1∶1000; HAF109, R&D Systems) in TBST containing 1% BSA for 1 h at 37°C. After washing with TBST, the reaction was visualized using an ECL Western Blotting Detection kit (GE Healthcare). The immunoblots signals were measured using Light Capture (ATTO, Tokyo, Japan). The target signal levels were normalized to β-actin.

### Subcellular fractionation

Subcellular fractioning was performed using ProteoExtract Subcellular Proteome Extraction kit (Merck KGaA, Darmstadt, Germany) according to the manufacturer’s protocol. Fractions of cytosol, membrane/organelle, nucleus and cytoskeleton were separated successively from fresh frozen liver. The cytosolic and nuclear fractions were processed by western blotting with anti-Nrf2. The purity of each fraction was determined by western blotting with anti-Calpain or anti-Histone H1 for the cytosolic and nuclear fractions, respectively.

### Immunohistochemistry

Frozen sections (6 µm in thickness) were cut using a cryostat and thaw-mounted onto silane-coated slides.

For fluorescence immunohistochemistry, the sections were incubated in 10 mM sodium citrate buffer (pH 6.0) for 10 min at 120°C (in autoclave), followed by incubation with anti-Nrf2 (10 µg/ml in 0.1 M PBS containing 5% normal goat serum and 0.3% Triton X-100). After rinsing twice with PBS, sections were incubated with the secondary antibody (biotinylated goat anti-rabbit IgG, Vector Laboratories) diluted 1∶200 in PBS for 1 h at 37°C. Finally, the sections were incubated in Texas-Red Avidin D (1∶1000; Vector Laboratories) in 0.1 M PBS containing 5% normal goat serum and 0.3% Triton X-100 for 1 h at 37°C, followed by nuclear staining with DAPI (Dojindo, Kumamoto, Japan). For non-fluorescent immunohistochemistry, sections were incubated with 3% H_2_O_2_ in distilled water for 20 min to quench the endogenous peroxidase activity. After rinsing twice with PBS, they were incubated with anti-HO-1 (1∶1000) primary antibody. After rinsing twice with PBS, sections were incubated with the secondary antibody (biotinylated goat anti-rabbit IgG, Vector Laboratories) diluted 1∶200 in PBS for 1 h at 37°C. After rinsing twice with PBS, the sections were incubated with avidin-biotin-HRP complex (ABC Elite kit, Vector Laboratories) for 1 h at 37°C. After washing in 0.05 M Tris-HCl buffer, pH 7.6, immunoreactivity was visualized by incubation in 0.05 M Tris-HCl buffer, pH 7.6, containing 0.02% 3, 3’-diaminobenzidine tetrahydrochloride and 0.005% H_2_O_2_ for 2–5 min. Omission of the primary or secondary antibody completely eliminated all immunoreactive staining.

### Microarray analysis and pathway analysis

The analysis of RNA quality showed that the A260/A280 nm absorbance ratio of RNA samples used in this experiment consistently ranged from 1.8 to 2.0. The quality of purified RNAs was assessed by an Agilent 2100 Bioanalyzer using an RNA 6000 Nano Kit (Agilent Technologies). The samples in which RNA Integrity Number (RIN) scores were between 8 and 10 were used in microarray and real-time RT-PCR. An equal amount of RNA from three rats in each group (livers of rats 3 h after treatment with vehicle or lansoprazole) was pooled and used for microarray analysis as described elsewhere [21], [22]. Briefly, total RNA (100 ng) was reverse-transcribed using a T7 sequence-conjugated oligo dT primer. Concomitantly, we used the RNA Spike-In Kit One Color (Agilent) to adjust the microarray data. Synthesis, amplification, and labeling of complementary RNA (cRNA) with Cy3 dye were performed according to the manufacturer’s protocols. Prepared cRNA was added to a whole rat genome oligo DNA microarray version 3.0 (4×44K; Agilent). Hybridization was performed at 65°C for 17 h. After washing, fluorescence intensity was determined using a scanner (G2565BA; Agilent). The Cy3 signal intensities were quantified and analyzed by background subtraction, using Feature Extraction software ver. 10.7.1.1 (Agilent), and the data were normalized using GeneSpring GX11.5.1 (Agilent). We used GeneSpring GX11.5.1 to select 12,134 genes producing florescence intensities > 100 in RNA samples from the livers of vehicle or lansoprazole treated rats.

We used Ingenuity Pathway Analysis (IPA; version Fall 2013) to determine the functional pathways of the identified genes. IPA software contains a database of biological interactions among genes and proteins, which was used to calculate the probability of a relationship between each canonical pathway and the identified genes. IPA scans the set of input genes to identify networks using Ingenuity Pathway Knowledge Base (IPKB) for interactions between identified ‘Focus Genes’, (in this study, the differently expressed genes between the livers treated with vehicle or lansoprazole) and known and hypothetical interacting genes stored in the IPA software. The data obtained was used to generate a set of networks with a maximum network size of 35 genes/proteins. Networks are displayed graphically as genes/gene products (‘nodes’) and the biological relationships between the nodes (‘edges’). All edges are from canonical information stored in the IPKB. In addition, IPA computes a score for each network according to the fit of the user’s set of significant genes. The score indicates the likelihood of the Focus Genes in a network from Ingenuity’s knowledge base being found together due to random chance. A score of 3, the cutoff for identifying gene networks, indicates that there is only a 10^−3^ chance that the locus genes shown in a network are due to random chance. Therefore, a score of 3 or higher indicates a 99.9% confidence level for excluding random chance.

### Data analysis

Statistical analysis was performed using one-way ANOVA followed by Fisher’s protected least significant difference test, or Student’s t-test using StatView software (Abacus Concepts, Berkeley, CA, USA).

## Results

### Up-regulation and nuclear translocation of Nrf2 in the liver following single oral treatment with lansoprazole

**Figure 1:**
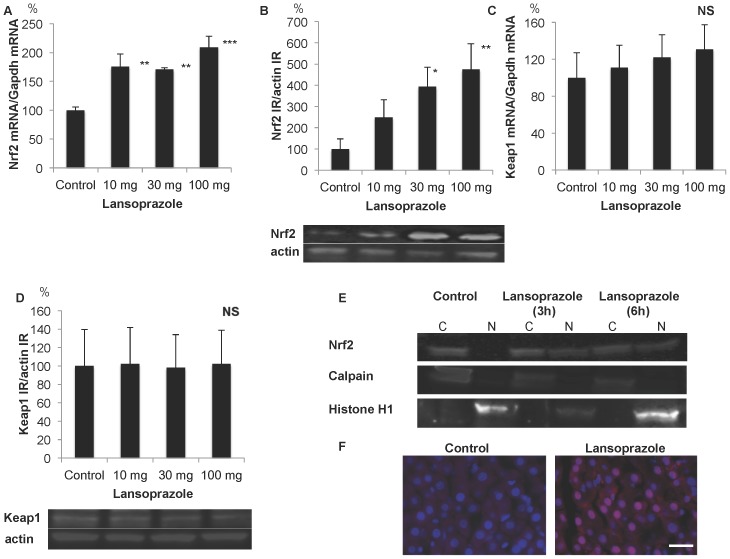
Up-regulation and nuclear translocation of Nrf2 in the liver following single oral treatment with lansoprazole. A) Up-regulation of Nrf2 mRNA in the liver at 3 h. ** P<0.01, ***P<0.001 compared with control. B) Up-regulation of Nrf2 immunoreactivity (IR) in the liver at 6 h. *P<0.05, ** P<0.01 compared with control. Representative photographs showing an increase of Nrf2 IR. C) No significant changes in Keap1 mRNA levels in the liver at 3 h. D) No significant changes in Keap1 IR levels in the liver at 6h. Representative photographs showing no change of Keap1 IR. E) Nuclear translocation of Nrf2 in the liver demonstrated by western blotting of cytosol fraction (C; Calpain-positive) and nuclear fraction (N; Histone H1-positive) at 3h and 6h. F) Nuclear translocation of Nrf2 in hepatocytes as demonstrated by double fluorescence immunohistochemistry. Blue indicates DAPI-positive nuclei, red indicates Nrf2 IR, and pink indicates the nuclear localization of Nrf2. Bar = 20 µm.

The levels of Nrf2 mRNA were increased at 3 h in a dose-dependent manner, with a 2-fold increase observed at 100 mg/kg, as compared to control levels (Figure 1A). The levels of Nrf2 IR in hepatic lysates were increased at 6 h in a dose-dependent manner, with a 5-fold increase observed at 100 mg/kg, as compared to control levels (Figure 1B). Conversely, the levels of Keap1 mRNA and IR were unchanged following treatment with lansoprazole (Figures 1C and 1D). Nuclear translocation of Nrf2 IR in hepatocytes was demonstrated using western blotting (Figure 1E) and immunohistochemistry (Figure 1F).

### Up-regulation of mRNA for Nrf2-dependent antioxidant and phase II enzymes following single oral treatment with lansoprazole

**Figure 2:**
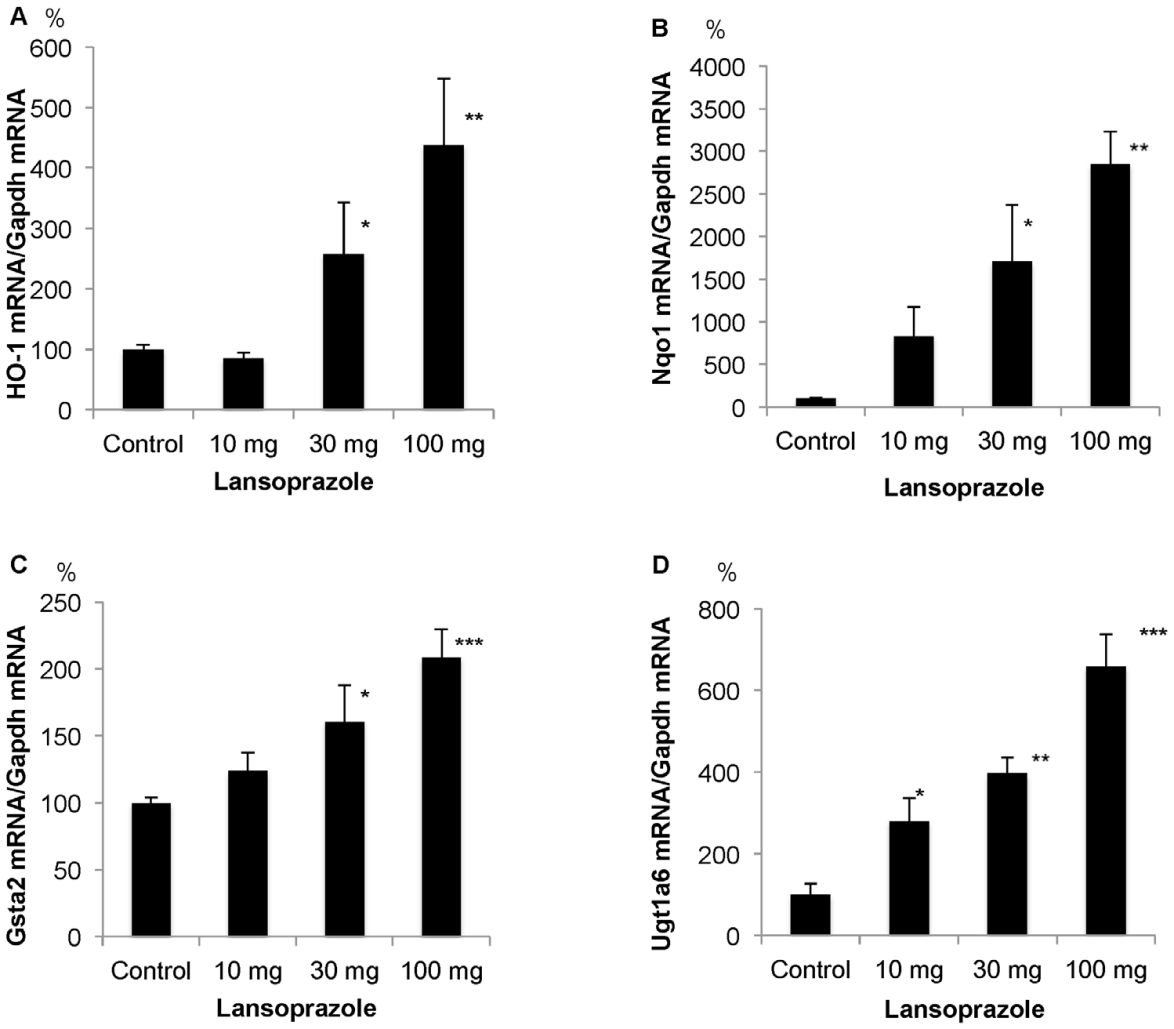
Up-regulation of mRNA for Nrf2-dependent antioxidant and phase II enzymes following single oral treatment with lansoprazole. A) Up-regulation of HO-1 mRNA in the liver at 3 h. B) Up-regulation of NAD (P) H dehydrogenase, quinone 1 (Nqo1) mRNA in the liver at 3 h. C) Up-regulation of glutathione S-transferase A2 (Gsta2) mRNA in the liver at 3 h. D) Up-regulation of UDP glucuronosyltransferase 1 family polypeptide A6 (Ugt1a6) mRNA in the liver at 3 h. *P<0.05, ** P<0.01, ***P<0.001 compared with control.

As shown in Figure 2, expression of mRNAs for Nrf2-dependent antioxidant and phase II enzymes such as HO-1, NAD (P) H dehydrogenase, quinone 1 (Nqo1), glutathione S-transferase A2 (Gsta2), UDP glucuronosyltransferase 1 family polypeptide A6 (Ugt1a6) was up-regulated at 3 h in a dose-dependent manner.

### Up-regulation of HO-1 IR in the hepatocytes following single oral treatment with lansoprazole

**Figure 3:**
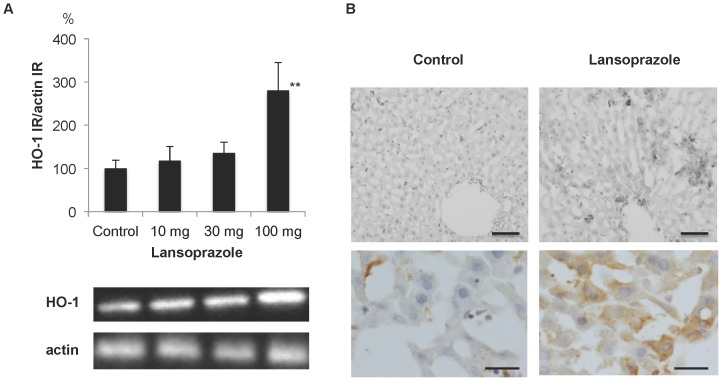
Up-regulation of HO-1 IR in hepatocytes following single oral treatment with lansoprazole. A) Up-regulation of HO-1 IR in the liver at 6 h. ** P<0.01 compared with control. Representative photographs showing an increase of HO-1 IR. B) Immunohistochemistry for HO-1 in the liver at 6 h. Signals for HO-1 IR were detected in macrophages in the control. *De novo* signals for HO-1 IR were observed in hepatocytes in response to treatment with lansoprazole. Upper panel bar = 100 µm. Lower panel bar = 20 µm.

The levels of HO-1 IR in the liver were increased at 6 h in a dose-dependent manner, with a 3-fold increase observed at 100 mg/kg, as compared to control levels (Figure 3A). In the control liver, HO-1 IR was detected in macrophages. In response to lansoprazole treatment, HO-1 IR positive hepatocytes were observed (Figure 3B).

### Up-regulation of Nrf2 and HO-1 following successive subcutaneous treatment with lansoprazole

**Figure 4:**
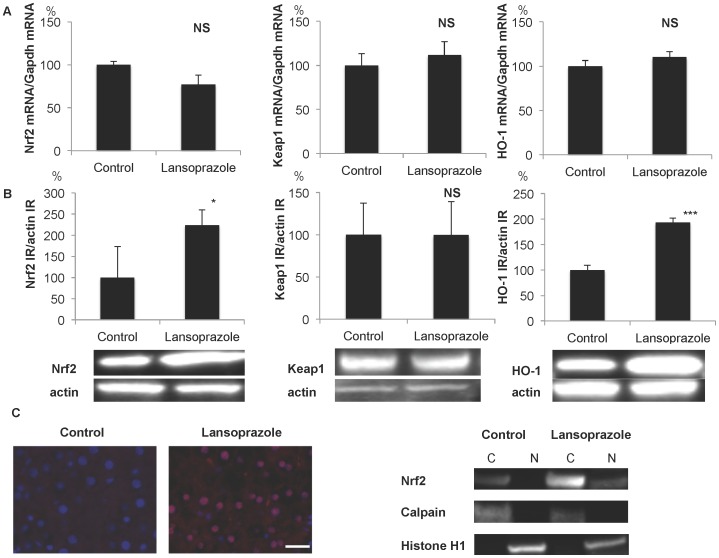
Up-regulation of Nrf2 and HO-1 following successive subcutaneous treatments with lansoprazole. A) No significant changes in the levels of mRNA for Nrf2, Keap1 and HO-1. B) Up-regulation of IR levels for Nrf2 and HO-1, with no change of Keap1 IR. *P<0.05, ***P<0.001 compared with control. Representative photographs showing an increase of Nrf2 and HO-1 IR. C) Nuclear translocation of Nrf2 in hepatocytes as demonstrated by double fluorescence immunohistochemistry and western blotting of cytosol fraction (C; Calpain-positive) and nuclear fraction (N; Histone H1-positive). Blue indicates DAPI-positive nuclei, red indicates Nrf2 IR, and pink indicates the nuclear localization of Nrf2. Bar = 20 µm.

As shown in Figure 4A, the levels of mRNA for Nrf2, Keap1 and HO-1 were not significantly different between control and animals receiving lansoprazole (30 mg/kg/day) for 7 successive days. However, the levels of IR for Nrf2 and HO-1 were significantly (2-fold) increased following administration of lansoprazole (Figure 4B). Nuclear translocation of Nrf2 in hepatocytes was also observed in response to treatment with lansoprazole (Figure 4C).

### Effects of successive subcutaneous treatment with lansoprazole on acute hepatic damage

**Figure 5:**
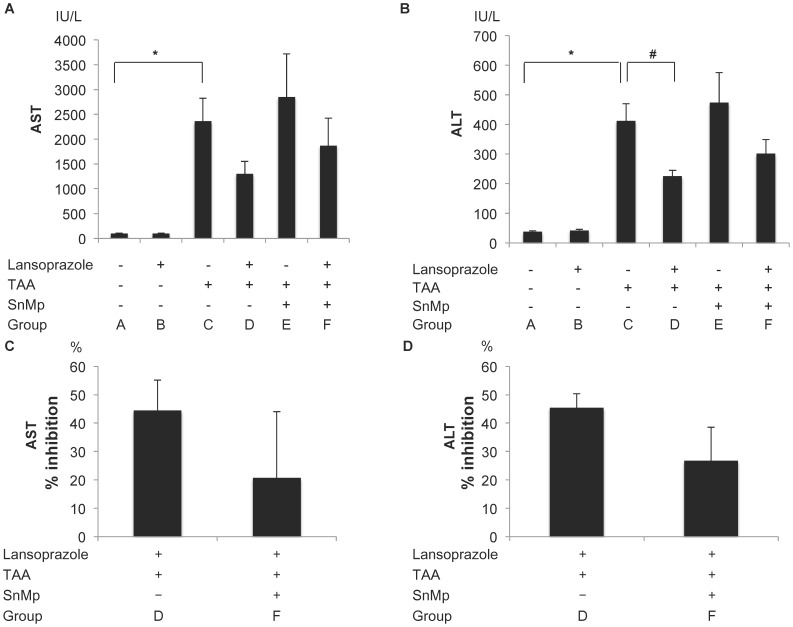
Serum levels and the % inhibition of AST and ALT in TAA-induced acute hepatic damage. A) Serum AST levels. * P<0.001 compared with group A (control). B) Serum ALT levels * P<0.0001 compared with group A (control). # P<0.01 compared with group C (acute hepatic damage). C) The % inhibition of TAA-induced increase of serum AST levels between group D (acute hepatic damage with lansoprazole) and group F (acute hepatic damage with lansoprazole and SnMP). D) The % inhibition of TAA-induced increase of serum ALT levels between group D (acute hepatic damage with lansoprazole) and group F (acute hepatic damage with lansoprazole and SnMP).

Treatment with lansoprazole (comparison between group A and group B) did not affect the serum levels of AST and ALT (Figure 5). In comparison to vehicle, treatment with TAA (comparison between group A and group C) produced a dramatic and significant increase in serum levels of AST (control, 85.0±21.7 IU/L vs. acute hepatic damage, 2358±471 IU/L, p<0.01) and ALT (control, 39.6±3.4 IU/L vs. acute hepatic damage, 436.5±39.4 IU/L, p<0.0001), indicating that TAA induced acute hepatic damage. Treatment with lansoprazole during acute hepatic damage, compared with acute hepatic damage alone (comparison between group C and group D) attenuated the increased serum levels of ALT (acute hepatic damage 2358±471 vs. acute hepatic damage with lansoprazole, 1300±253, P = 0.11) and ALT (acute hepatic damage, 436.5 ±39.4 vs. acute hepatic damage with lansoprazole, 224.2±20.1, P<0.01), indicating that lasoprazole protects against acute hepatic damage. Treatment with SnMP during acute hepatic damage compared with acute hepatic damage alone (comparison between group C and group E) slightly augmented the elevated serum levels of AST (acute hepatic damage 2358±471 vs. acute hepatic damage with SnMP, 2851±868, P = 0.44) and ALT (acute hepatic damage 436.5±39.4 vs. acute hepatic damage with SnMP, 474.3±102.1, P = 0.58), suggesting that native HO-1, mainly expressed in the hepatic macrophages, may have a slight protective effect against acute hepatic damage. Treatment with a combination of lansoprazole and SnMP, compared with lansoprazole alone (comparison between group D and group F) slightly augmented the increased serum levels of AST (acute hepatic damage with lansoprazole 1300±253 vs. acute hepatic damage with lansoprazole and SnMP, 1871±551, P = 0.39) and ALT (acute hepatic damage with lansoprazole 224.3±20.1 vs. acute hepatic damage with lansoprazole and SnMP, 301.3±48.3, P = 0.26), suggesting that the induction of HO-1 in hepatocytes may partially ameliorate acute hepatic damage. Treatment with lansoprazole and SnMP during acute hepatic damage compared with acute hepatic damage alone (comparison between group C and group F) slightly attenuated the increased serum levels of AST (acute hepatic damage alone 2358±471 vs. acute hepatic damage with lansoprazole and SnMP, 1871±551, P = 0.45) and ALT (acute hepatic damage alone 436.5±39.4 vs. acute hepatic damage with lansoprazole and SnMP, 301.3±48.3, P = 0.056), suggesting that the induction of anti-oxidative enzymes, excluding HO-1, in hepatocytes may partially contribute to the amelioration of acute hepatic damage. The % inhibition in group D (acute hepatic damage with lansoprazole) was two-fold higher than that in group F (acute hepatic damage with lansoprazole and SnMP) (AST; 44.5±10.6% vs. 20.7±23.4, P = 0.39, ALT; 45.4±4.9 vs. 26.7±11.8, P = 0.19), suggesting that the effect of lansoprazole on the acute hepatic damage is mediated by via both HO-1-dependent and HO-1-independent pathways (Figures 5C and 5D).

**Figure 6:**
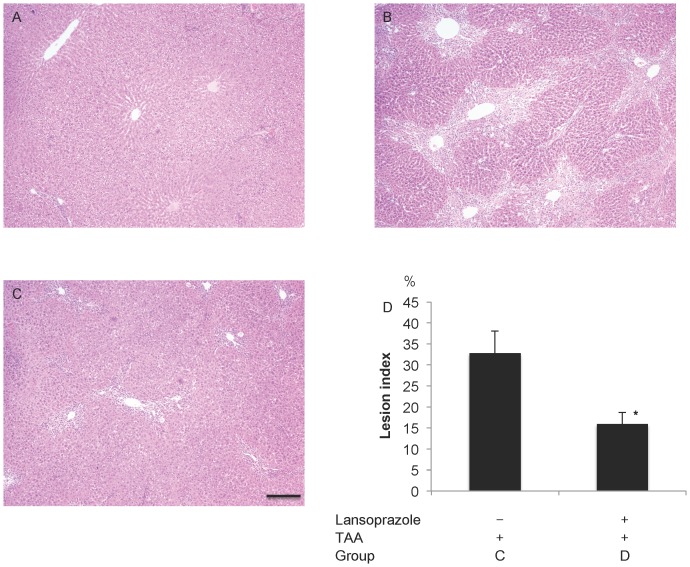
Histology of the liver stained with hematoxylin-eosin. A) Control (group A). B) TAA-treated liver (group C). C) TAA-treated liver pretreated by lansoprazole (group D), Bar = 250 µm. D) Lesion index (%). * P<0.05.

Hepatic damage was also assessed using histological examination. Following TAA-treatment, hepatocellular degeneration and necrosis with hemorrhage and infiltration of inflammatory cells were observed around the central vein (Figure 6). In response to pretreatment with lansoprazole, these pathological changes were significantly attenuated (acute hepatic damage; 32.8±5.3% vs. acute hepatic damage with lansoprazole; 15.9±2.8%, P<0.05).

### Microarray analysis and data mining by IPA

We investigated liver gene expression profiles 3 h after treatment with lansoprazole (100 mg/kg) using microarray analysis. Among the 30,367 genes that were analyzed, 12,134 genes were detected in the lansoprazole- and/or vehicle-treated livers. We selected genes whose expression differed by more than 2-fold in the lansoprazole group compared with the vehicle group. Using these criteria, we identified 1874 up-regulated genes and 1,700 down-regulated genes in the lansoprazole group compared to the vehicle group.

**Figure 7:**
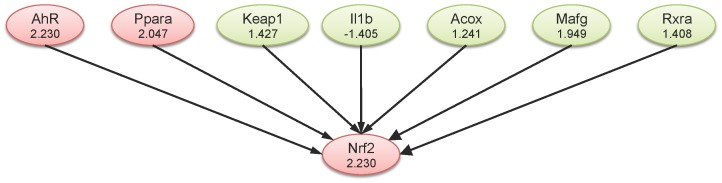
Up-stream networks of Nrf2 in the liver at 3 h following oral administration of lansoprazole (100 mg/kg) in the liver. The number below each symbol indicates fold change observed in the microarray analysis. Pink color indicates significant up-regulation, and green color indicates no significant change in gene expression. Acox1, acyl-CoA oxidase 1, palmitoyl; Ppara, peroxisome proliferator activated receptor α; IL1β, interleukin 1β, Mafg, v-maf avian musculoaponeurotic fibrosarcoma oncogene homolog G; Rxra, retinoid X receptor α.

IPA was used to organize the differentially expressed genes into functionally annotated pathways and networks. Using IPA, we identified 3 networks with scores greater than 20 (Table 2). IPA indicated that 7 genes (Acox1, AhR, Pparα, Keap1, IL1β, Mafg and Rxra) were identified as the upstream of Nrf2 (Figure 7). Within these genes, AhR and Pparα (peroxisome proliferator activated receptor α) were significantly increased (ratio>2).

**Table 2:** Lansoprazole inducing networks.

ID	Molecules in Network	Score	Focus Molecules	Top Functions
1	ABCB1, ABCD2, ACACA, Acot1, AKR1B1, AR, ASNS, CCNT1, CDC6, CEBPB, CTSE, CYP2B6, CYP7A1, ELOVL3, ELOVL5, ELOVL6, ESRRG, FGF21, HMGCR, IKBKG, INSIG1, LPIN1, NR0B2, NR1I3, NR5A2, PHLDA1, PPARA, PPARGC1A, RNF186, SELENBP1, STBD1, SULT2A1, TLR5, TOP2A, TRIB3	33	35	Lipid Metabolism, Molecular Transport, Small Molecule Biochemistry
2	ABCC3, ADAM8, AGER, CCL4, CCL13, CCNA1, CD38, CD44, CD86, CEACAM1, CELA1, CLDN7, CLEC7A, CXCR3, GAB2, GSTA1, IFNG, IL6, IL1R1, IL1R2, INSR, IRF8, ITGAM, Ly6a (includes others), MAFF, MAFK, MAPK8, MMP9, NCAM1, NLRP3, PTPN11, SERPINE1, TLR3, TLR7, TXNRD1	33	35	Cell-To-Cell Signaling and Interaction, Cellular Movement, Hematological System Development and Function
3	AHR, ATF7, CAMK2N2, CCRN4L, Cml5, COL27A1, CYP1A1, CYP1A2, CYP1B1, CYP26A1, DCLK3, ENTPD5, GCLM, GSR, GSTA5, Gstm3, HMOX1, LPL, MARCO, MID1, MT4, NFE2L2, NFIA, NQO1, Pfn2, Rdh1 (includes others), RGS16, SERPINA12, SHANK2, SIDT2, SLC46A3, SRXN1, TGFB2, THRSP, TRPM8	23	30	Lipid Metabolism, Small Molecule Biochemistry, Vitamin and Mineral Metabolism
4	ABCC4, ABCG8, ACTG1, AKT2, Aldh1a7, CLDN3, CYP2B6, CYP7A1, DIO1, ELOVL2, ELOVL5, ELOVL6, GADD45B, Gstm3, GSTM5, IGF1R, Kap, KRT19, MAFF, MAP3K5, MDM2, MRGPRX3, MT1E, NEDD9, NR1H4, NR1I3, PAX8, POR, PPP1R16A, SCD, SLC13A1, sphingomyelin, UGT2B4, UPP2, ZNF292	13	23	Lipid Metabolism, Small Molecule Biochemistry, Molecular Transport,
5	ACVR1, ADAMTS5, ADH4, BCL2, CASP3, CCND1, CENPA, CSF1, CTGF, CXCL3, CYP2B6, CYP4A22, EGLN3, ELOVL3, ethanol, FADD, FASLG, GADD45A, GPD1, GPT, GSTA5, Hamp/Hamp2, IL1B, IL1RN, ITGAV, MMEL1, NCF1, NQO1, PLK3, PTGS2, RHOB, SLC7A11, TLR9, UBE2V2, YES1	11	21	Cell Death and Survival, Liver Necrosis/Cell Death, Cellular Development

**Figure 8:**
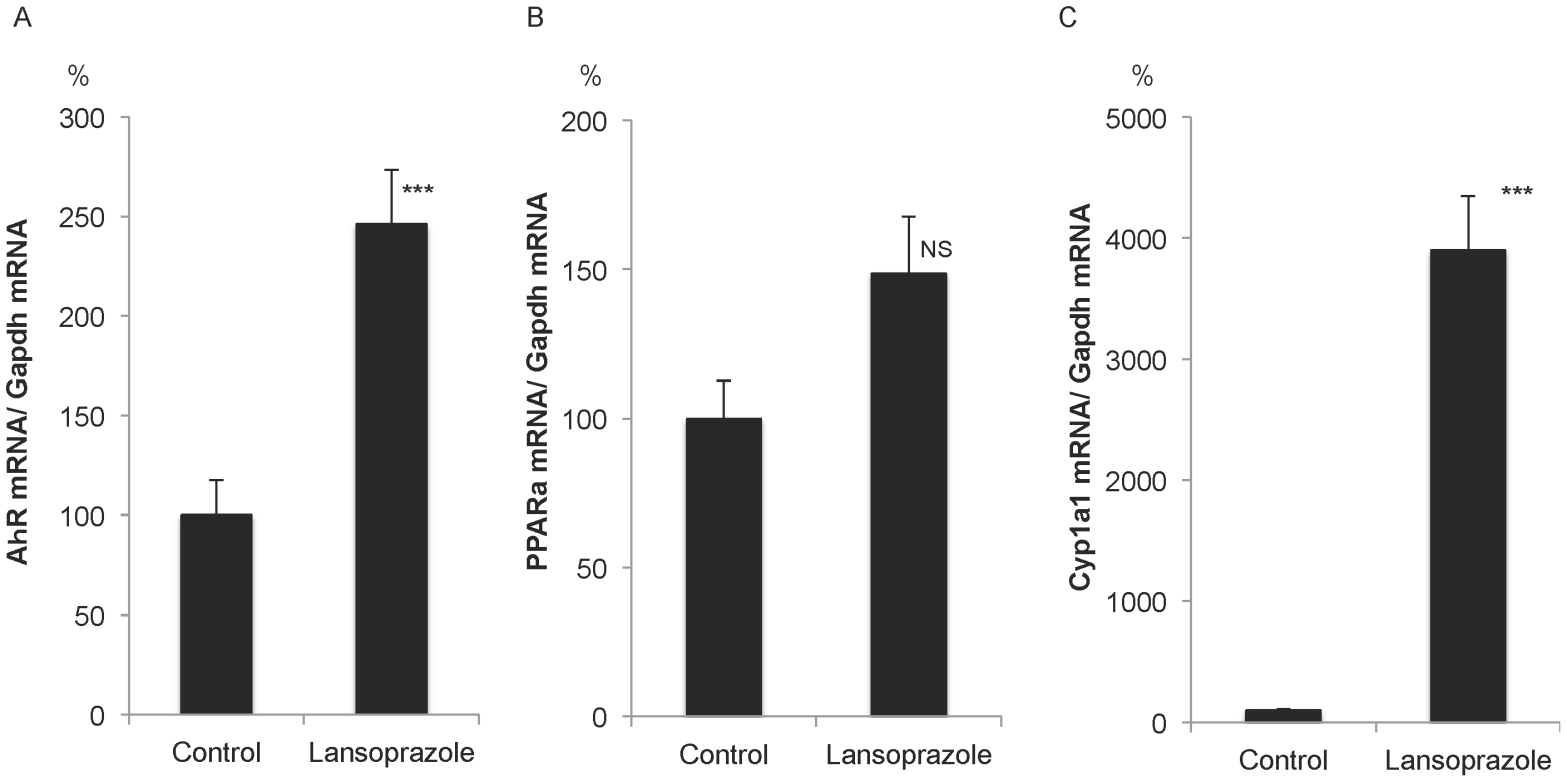
Validation of gene expression changes in the liver at 3 h following oral administration of lansoprazole (100 mg/kg). A) Up-regulation of AhR mRNA. ***P<0.0001. B) No significant change (P = 0.068) of Pparα mRNA levels. C) Up-regulation of Cyp1a1 mRNA. ***P<0.0001.

To confirm these networks, the expression of these genes was evaluated using real-time RT-PCR (Figure 8). The mRNA level of AhR was significantly increased. Expression of Pparα was elevated in a non-significant manner. The mRNA level of Cyp1a1, a target gene of AhR, was significantly and markedly up-regulated.

## Discussion

Lansoprazole is available worldwide as a potent proton pump inhibitor. In this study, we identified a novel acid-independent, extra-gastrointestinal function as a potent activator of anti-oxidative stress responses. First, we found that gastric or subcutaneous administration of lansoprazole induced the transcription of Nrf2 mRNA and the translation of Nrf2 protein without affecting Keap1 levels in the liver. Second, un-complexed Nrf2 was translocated to the hepatic nuclei, thereby initiating the transcription of Nrf2-dependent antioxidant and phase II enzymes, such as HO-1, Nqo1, Gsta2 and Ugt1a6, in the liver. Third, pretreatment with lansoprazole attenuated TAA-induced acute hepatic damage via both HO-1-dependent and HO-1-independent pathways. Fourth, the AhR-Cyp1a1 pathway was associated with the up-regulation of Nrf2 mRNA.

Induction of HO-1 by proton pump inhibitors was first reported by Becker et al [5], who found that both lansoprazole and omeprazole up-regulated the mRNA, IR and activity of HO-1 in human gastric cancer cells (AGS cells and KATO cells), RGM-1 cells and human endothelial cells (ECV304). Lansoprazole but not omeprazole induced HO-1 IR at the surface of the small intestinal epithelial cells and prevented indomethacin-induced small intestinal ulceration [7]. In our preliminary study, the induction of HO-1 mRNA in the small intestine by lansoprazole was not observed at 3 h. Although we missed the peak of mRNA induction, the mechanism of increased HO-1 IR in the small intestine might not be associated with the induction of Nrf2 mRNA. Discrepancies between the levels of mRNA and those of IR were also observed in the levels of Nrf2 and HO-1 following successive subcutaneous treatment with lansoprazole. This might be also due to the delay in sampling causing some degradation of induced Nrf2 and HO-1 mRNAs. Using RGM-1 cells, it was also demonstrated that phosphorylation of extracellular signal-regulated kinase (ERK) and Nrf2, as well as the activation and nuclear translation of Nrf2 and oxidation of Keap1, were involved in lansoprazole-induced HO-1 up-regulation [6]. A similar mechanism could not be excluded in the liver. However, in contrast, the mechanism of HO-1 induction by lansoprazole in the liver was considered to be due to *de novo* Nrf2 mRNA synthesis without affecting the levels of Keap1, rather than due to the dissociation of the Keap1-Nrf2 complex.

Lansoprazole is metabolized by Cyp2c19 and Cyp3a4 [23]. Cyp3a4 catalyzes both 5-hydroxylation and sulfoxidation of lansoprazole and Cyp2c19 catalyzes 5-hydroxylation in the human liver. In contrast, lansoprazole, omeprazole, and pantoprazole induced Cyp1a1, Cyp1a2, Cyp2b and Cyp3a in primary human hepatocytes, human liver, human hepatoma cells and rat liver [24]–[26]. AhR is involved in the induction of Cyp1a1 and Cyp1a2 by omeprazole via a common regulatory region containing multiple AhR-binding motifs [26]. Our study has confirmed the extensive induction of Cyp1a1 in rat liver by lansoprazole. Complex mutual interactions between AhR and Nrf2 occur during the induction of phase I and phase II drug-metabolizing enzyme genes, exemplified by the mutual induction of gene expression. AREs are present in many phase II genes, whereas the xenobiotic response elements (XRE) are present in both phase I genes and phase II genes [27]. The AhR and AhR nuclear translocator (ARNT) heterodimer binds to XRE, resulting in the induction of both phase 1 genes and Nrf2, with Nrf2 subsequently activating phase II genes [28]. Conversely, Nrf2 regulates the expression of AhR mRNA and subsequently modulates several downstream genes in the AhR signaling pathway, including transcriptional control of phase 1 genes (Cyp1a1 and Cyp1b1) [29]. 2,3,7,8-Tetrachlorodibenzo-*p*-dioxin (TCDD) binds to the AhR, and this complex translocates to the nucleus [30]. Via activation of the AhR/XRE system, TCDD induces Cyp1a1, Nqo1, Ugt1a6 and Gsta1 as well as Nrf2 in the liver [31]. In contrast, most of the TCDD-induced enzymes, excluding Cyp1a1 and Ugt1a1, are not induced in the Nrf2-null mice [31]. In this study, we found that lansopazole was a mixed inducer of both phase I and phase II drug-metabolizing systems, i.e. the mRNA levels of AhR, Cyp1a1, Nrf2, and phase II enzymes. The molecular mechanism of these complex interactions remains to be elucidated.

The other pathway in relation to the induction of Nrf2 mRNA by lansoprazole is the Pparα pathway, although its contributions are limited. The absence of the Pparα gene results in down-regulation of Nrf2 in the liver of fasted animals [32]. These observations may be related to lansoprazole-induced Nrf2 expression.

**Figure 9:**
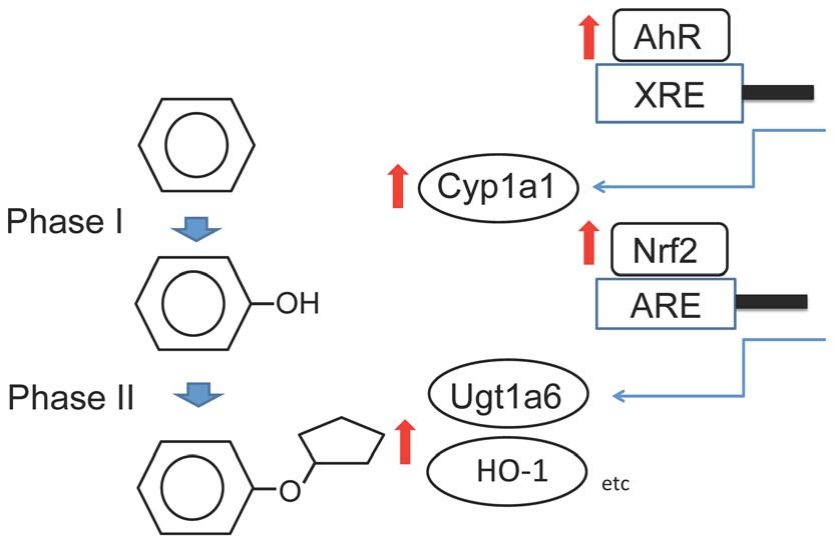
A summary schematic of the lansoprazole-induced AhR/Cyp1a1/Nrf2 pathway in the liver. XRE, xenobiotic response elements; ARE, antioxidant response element.

A summary schematic of the lansoprazole-induced AhR/Cyp1a1/Nrf2 pathway in the liver is shown in Figure 9.

### Study limitations

In this study, we first determined mRNA levels at 3 h and IR at 6h after gastric administration of lansoprazole. Since we did not examine the expressions of these substances at other time points, it is possible that we overlooked peak expression levels of each substance. In previous studies, we performed detailed estimates of the time course of HO-1 induction by acute gastric injury [33] and polaprezinc [20]. These studies indicated that HO-1 mRNA levels peaked at 3h and HO-1 IR levels peaked at 6 h. To minimize the number of animals used in this study, we selected sampling points and the minimal replicate numbers required to achieve statistical significance. Second, the dosages of lansoprazole (10–100 mg/kg) were higher than those used in clinical settings. We selected dosages that were used in several experimental studies involving rat ulcer models [34]. Third, there are no reports of the effects of lansoprazole on hepatic function in human, specifically during hepatic dysfunction. Clinical assessments of lansoprazole in hepatic diseases are currently in progress in our group. Fourth, the effects of other proton pump inhibitors have not yet been examined. It has been reported that lansoprazole, but not omeprazole, induced HO-1 in gastric mucosal cells [6] and the small intestine [7]. In contrast, another study demonstrated that both lansoprazole and omeprazole induced HO-1 in gastric and endothelial cells [5]. In addition, proton pump inhibitors (including omeprazole, lansoprazole and pantoprazole) were found to induce Cyp1a1, Cyp1a2, Cyp2b and Cyp3a in various types of hepatocytes [24]–[26]. These effects were observed in cultured cells, suggesting that the effects are independent of gastric acid suppression. It is reasonable to consider that up-regulation of the AhR/Cyp1a1/Nrf2/phase II system by lansoprazole is also independent of gastric acid suppression. Microarray analysis showed that the levels of mRNA encoding the proton pump H^+^/K^+^-adenosine triphosphatase were below the detection limit (data not shown), suggesting that a functionally active proton pump is not expressed in the liver. Therefore, it is highly unlikely that this novel effect of lansoprazole is attributable to proton pump inhibition. We are investigating a number of chemical structures within lansoprazole that may contribute to the up-regulation of the AhR/Cyp1a1/Nrf2/phase II system in the liver. Extensive screening of a chemical library, not limited to proton pump inhibitors, is also in preparation by our group. Fifth, the precise molecular mechanisms by which lansoprazole activates both the phase I and phase II drug-metabolizing systems, i.e., the AhR/Cyp1a1/Nrf2 pathway, are unknown. Studies in Nrf2-null mice may provide a possible cue to answer these questions [31]. Sixth, anti-oxidative stress effects on other organs have not been clarified. Further extensive studies are required to answer these questions.
